# mTOR pathway activation is a favorable prognostic factor in human prostate adenocarcinoma

**DOI:** 10.18632/oncotarget.8767

**Published:** 2016-04-16

**Authors:** Suzan Stelloo, Joyce Sanders, Ekaterina Nevedomskaya, Jeroen de Jong, Dennis Peters, Geert J.L.H. van Leenders, Guido Jenster, Andries M. Bergman, Wilbert Zwart

**Affiliations:** ^1^ Division of Molecular Pathology, The Netherlands Cancer Institute, Amsterdam, The Netherlands; ^2^ Division of Pathology, The Netherlands Cancer Institute, Amsterdam, The Netherlands; ^3^ Department of Pathology, Josephine Nefkens Institute, Erasmus Medical Center, Rotterdam, The Netherlands; ^4^ Department of Urology, Josephine Nefkens Institute, Erasmus Medical Center, Rotterdam, The Netherlands; ^5^ Division of Medical Oncology, The Netherlands Cancer Institute, Amsterdam, The Netherlands

**Keywords:** mTOR, PI3K pathway, ERG, prostate cancer, prognosis

## Abstract

Prostate cancer patients with localized disease are treated with curative intent. However, the disease will recur in approximately 30% of patients with a high incidence of morbidity and mortality. Prognostic biomarkers are needed to identify patients with high risk of relapse. mTOR pathway activation is reported in prostate cancer, but clinical trials testing efficacy of mTOR inhibitors were unsuccessful. To explain this clinical observation, we studied the expression and prognostic impact of mTOR-S2448 phosphorylation in localized prostate carcinomas. mTOR-S2448 phosphorylation is indicative for an activated mTOR pathway in prostate cancer. Surprisingly, the mTOR signaling pathway is activated specifically in prostate cancer patients with a favorable outcome. Since tumors from poor-outcome patients have low levels of mTOR-S2448 phosphorylation, this may explain why mTOR inhibitors proved unsuccessful in prostate cancer trials.

## INTRODUCTION

Prostate cancer is the second most common malignancy in men worldwide [1]. Current diagnostic methods for prostate cancer include serum concentration of prostate specific antigen (PSA) measurement and digital rectal examination (DRE), often followed by transrectal ultrasound-guided biopsies for histological determination [2]. These tools are used for treatment selection and prognostication. However, the parameters gathered by the above mentioned methods (e.g. PSA serum level, number of positive biopsies, Gleason and TNM stage) are insufficient for reliable estimation of disease-free survival and optimal treatment selection [3–5]. There is a pressing clinical need for prognostic markers to distinguish the patients with a low-risk from those with a high-risk of relapse. Such prognostic factors would not only prevent overtreatment, but also identify those patients who may benefit from additional therapies.

The Phosphoinositide 3-kinase (PI3K)- AKT- mammalian target of rapamycin (mTOR) pathway stimulates cell survival, growth and differentiation [6] and is often activated in prostate cancer [7, 8]. The activation of the PI3K-AKT signaling cascade results in the phosphorylation of mTOR (p-mTOR) at serine residue 2448 (S2448) [9], consequently phosphorylating downstream effectors, such as eukaryotic initiation factor 4E (eIF4E) and the ribosomal S6 kinase 1 (S6K1) [10]. In addition to AKT, S6K1 can also phosphorylate S2448 through a feedback loop of which the functional significance remains unclear [11, 12].

Patients with activated mTOR signaling in tumor cells are expected to benefit from treatment with mTOR inhibitors, such as everolimus, rapamycin and temsirolimus. mTOR inhibitors proved highly successful in prolonging progression-free survival in breast cancer and renal cancer, albeit with considerable side-effects [13–15]. However, in prostate cancer, mTOR inhibitors have demonstrated limited clinical efficacy in the castration resistant [16] and neoadjuvant setting [17]. Although mTOR inhibitors blocked mTOR signaling in prostate cancer, no effects on growth reduction, apoptosis and grade change were reported. A previous report on mTOR phosphorylation in prostate cancer identified a small subpopulation of p-mTOR negative patients who may benefit from mTOR inhibition by integrating mTOR phosphorylation, ERG fusion and PTEN mutation status [18]. Yet, this report could not be confirmed by others [19] and was in disagreement with multiple cell biological reports [9, 20].

In this report, we evaluate a potential correlation of mTOR pathway activation with biochemical relapse-free survival in primary prostate cancer. Since mTOR inhibitors particularly target tumors with an activated mTOR pathway, linking mTOR activity with outcome could potentially explain the poor performance of mTOR inhibitors in the treatment of prostate cancer.

## RESULTS

### mTOR phosphorylation and association with clinical parameters

**Figure 1:**
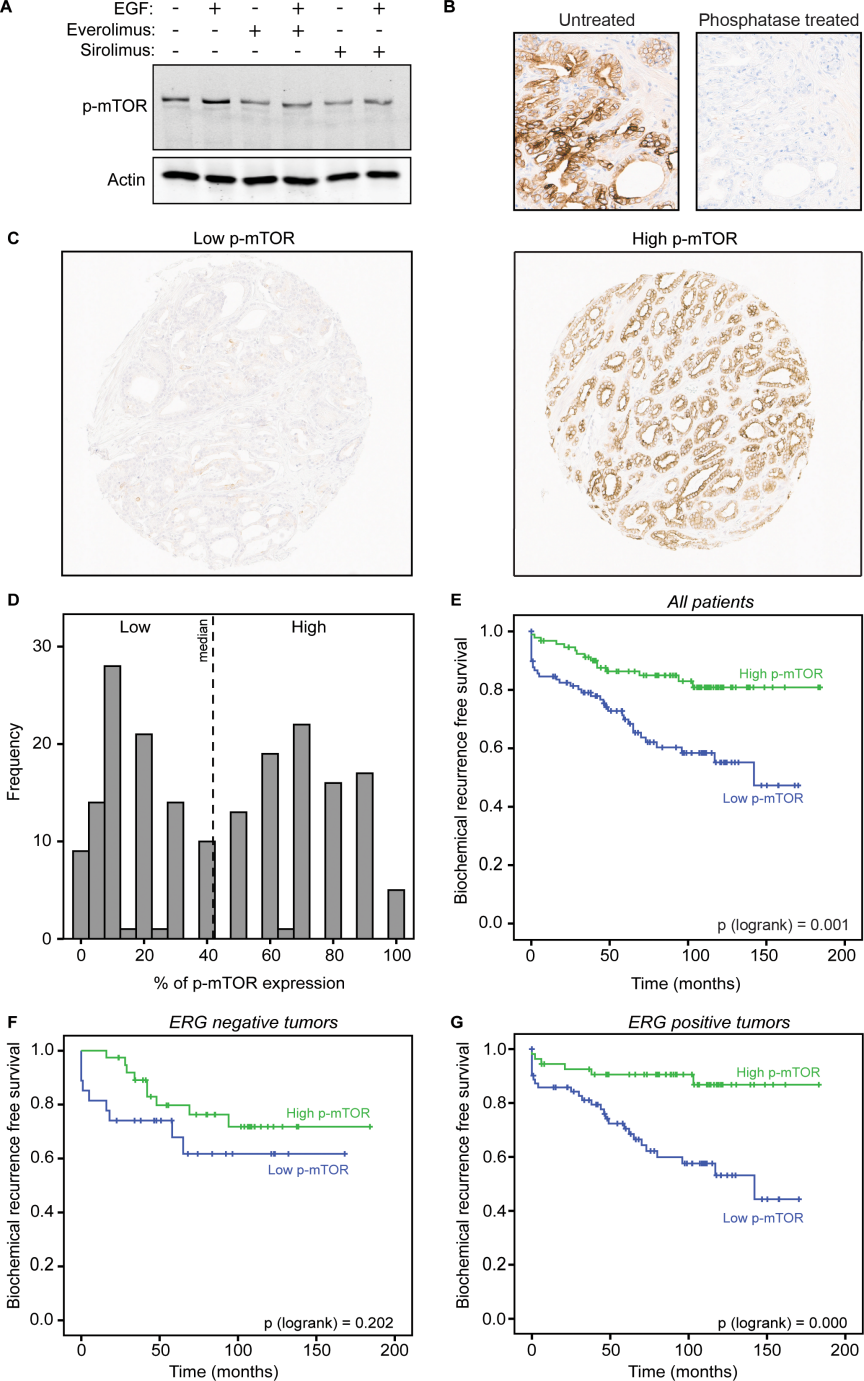
p-mTOR expression in tumor cells identifies prostate cancer patients with favorable outcome **A.** LNCaP cells were pretreated with vehicle, everolimus or sirolimus for 3 hours followed by EGF stimulation for 20 minutes. Western blot for p-mTOR is shown with actin acting as a loading control. **B.** Primary prostate cancer tissue was untreated or treated with phosphatase prior to staining for p-mTOR to confirm phospho-specificity of the antibody. **C.** Representative immunostaining of tissues with low and high percentage of tumor cells positive for p-mTOR in primary prostate cancer tissue. **D.** Bimodal distribution of p-mTOR immunoscoring. Dotted line indicates the median of positive p-mTOR scoring in tumor cells (%). **E.** Kaplan-Meier curves of biochemical recurrence free survival of the two groups of patients based on the median percentage of positive tumor cells, lower than 40% or higher than 40% p-mTOR positivity. **F.** Kaplan-Meier curves of biochemical recurrence free survival of patients with negative ERG expression grouped in either low p-mTOR or high p-mTOR expression. **G.** Kaplan-Meier curves of biochemical recurrence free survival of patients with positive ERG expression grouped in either low p-mTOR or high p-mTOR expression.

For immunohistochemical studies, tissue microarrays (TMAs) were used. From the original study (n = 481), phosphorylated mTOR at serine 2448 was evaluable for at least 2 tissue cores from 191 patients. Patient characteristics are shown in Table 1. In this cohort, no adequate p-mTOR expression could be assessed in 290 of the 481 patients (60%) due to absence of tumor cells in the cores and missing cores (clinical characteristics previously published [21]). Immunohistochemical analysis of the evaluable prostate cancer tissue demonstrated submembranous p-mTOR staining in 182 cases (95%), negative staining in 9 cases (5%) or at least 1 core negative in 36 cases (19%). Antibody specificity was confirmed with western blotting, showing a single band of the expected molecular weight (289KDa) with induced signal after EGF stimulation and decreased signal after mTOR inhibition by everolimus and sirolimus treatment (Figure 1A). Additionally, p-mTOR staining of prostate tissue was completely eliminated by lambda phosphatase treatment demonstrating that the antibody specifically recognized phosphorylated mTOR (Figure 1B). Representative p-mTOR immunostaining of low (5%) and high percentage (90%) of tumor cells expressing p-mTOR is shown in Figure 1C. For further analysis, patients were separated into two groups, low and high p-mTOR, based on the median percentage (40%) of positive tumor cells (Figure 1D). The relation between p-mTOR expression and clinico-pathological parameters is summarized in Table 2. Low p-mTOR expression is significantly associated with a higher pathologic T (pT) stage (p = 0.01). Furthermore expression of ERG, evaluated previously on this cohort [21], was more frequently observed in patients with low p-mTOR expression (p = 0.04). There was no significant relation between p-mTOR and Gleason score (p = 0.47), surgical margin status (p = 0.38), initial PSA level (p = 0.14) and age (p = 0.33).

**Table 1:** Clinico-pathological parameters

**Age (Years)**		**Surgical margins**	
Median	65	Positive	57 (29.8%)
Mean	65	Negative	134 (70.2%)
Min	56		
Max	75	**Lymph node metastasis**	
		Yes	0 (0%)
**PSA (ng/ml)**		No	191 (100%)
Median	6.0		
Mean	9.2	**Biochemical recurrence**	
Min	0.3	Yes	51 (26.7%)
Max	152.2	No	140 (73.3%)
**Gleason**		**Local recurrence**	
5	19 (9.9%)	Yes	13 (6.8%)
6	65 (34%)	No	178 (93.2%)
7	88 (46.1%)		
8	12 (6.3%)	**Overall death**	
9	7 (3.7%)	Yes	30 (15.7%)
		No	161 (84.3%)
**pT stage**			
T2	127 (66.5%)	**Death from prostate cancer**	
T3	54 (28.3%)	Yes	4 (2.1%)
T4	10 (5.2%)	No	70 (36.6%)
		Unknown	117 (61.3%)

**Table 2:** Relation of immunohistochemical p-mTOR expression with clinical pathological parameters

	Low p-mTOR	High p-mTOR	p-value
**n**	98	93	
**Age (Years)**	65.33	64.72	0.33
**PSA (ng/ml)**	10.75	7.51	0.14
**Gleason**			0.47
<7	39	45	
7	48	40	
>7	11	8	
**pT stage**			0.01
T2	56	71	
T3	34	20	
T4	8	2	
**Surgical margins**			0.38
Positive	32	25	
Negative	66	68	
**ERG expression**			0.04
Positive	71	54	
Negative	27	39	

### mTOR phosphorylation correlates with favorable outcome in prostate cancer

Next, p-mTOR expression was tested in relation to outcome. Patients with high p-mTOR had a low risk of biochemical recurrence development (HR = 0.36, p = 0.001, 95% CI 0.20 − 0.66) (Figure 1E). Pathological parameters PSA, Gleason score, pT-stage and surgical margins were also predictors of outcome in univariate analysis (p < 0.0001) (Table 3). Furthermore, high p-mTOR expression remained an independent predictor of biochemical recurrence free survival in multivariate analysis (HR = 0.45, p = 0.01, 95% CI 0.24-0.84) (Table 3). Also surgical margins and Gleason score remained significant predictors of outcome in multivariate analysis (p = 0.03 and 0.02, respectively).

**Table 3:** Uni- and multivariate Cox regression analysis

Variable	Univariate analysis		Multivariate analysis	
	HR (95% CI)	p-value	HR (95% CI)	p-value
Age (Years)	1.04 (0.98-1.11)	0.23	1.02 (0.95-1.09)	0.68
PSA	1.02 (1.01-1.03)	<0.0001	1.01 (1.00-1.03)	0.10
Gleason				
<7	Reference	<0.0001	Reference	0.02
7	2.97(1.52-5.82)	0.002	2.28 (1.12-4.65)	0.02
>7	6.27 (2.59-15.22)	<0.0001	3.78 (1.43-9.99)	0.04
pT stage				
T2	Reference	<0.0001	Reference	0.42
T3	2.01 (1.10-3.69)	0.03	1.06 (0.54-2.10)	0.86
T4	4.63 (2.08-10.33)	<0.0001	1.77 (0.72-4.39)	0.21
Surgical margin	2.97 (1.71-5.16)	<0.0001	2.03 (1.09-3.78)	0.03
ERG expression	0.91 (0.51-1.61)	0.74	0.88 (0.47-1.64)	0.70
p-mTOR	0.36 (0.20-0.66)	0.001	0.45 (0.24-0.84)	0.01

Previous data implicate mTOR-S2448 phosphorylation as indicative for good outcome in ERG-fusion prostate cancers [18]. We confirmed this finding using available ERG immunohistochemical data from the same cohort [21], showing that mTOR-S2448 phosphorylation correlated with a favorable outcome in ERG-positive cases (p < 0.0001), while this was not the case in ERG-negative tumors (p = 0.202) (Figure 1F–1G).

### mTOR-S2448 phosphorylation highlights an activated mTOR pathway in prostate cancer

**Figure 2:**
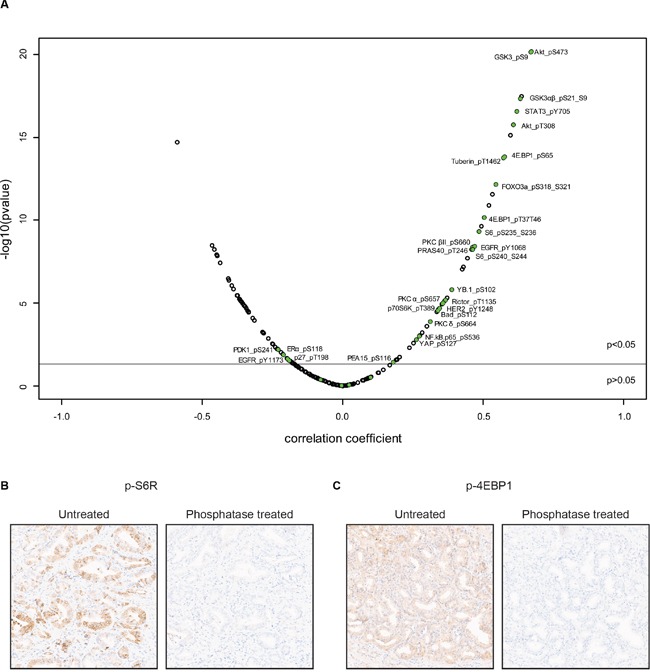
p-mTOR expression positively correlates with PI3K pathway members phosphorylation **A.** Volcano plot showing Pearson's coefficients for correlation of mTOR-S2448 phosphorylation with expression of 188 other proteins (list is provided in Supplementary Table S1). The Y-axis represents the −log10 of the p-value, adjusted for multiple testing. The horizontal line corresponds to p = 0.05. Phospho-proteins described to be involved in the PI3K pathway are colored in green. RPPA data was generated by TCGA Research Network [22]. **B, C.** Primary prostate cancer tissue was untreated or treated with phosphatase prior to staining for p-S6R (B) and p-4EBP1 (C) to confirm phospho-specificity of the antibody.

mTOR-S2448 phosphorylation can identify prostate cancer patients with a favorable outcome. But does mTOR-S2448 phosphorylation imply activation of both upstream and downstream signaling cascades in prostate cancer? To answer this, we analyzed reverse phase protein array (RPPA) data from 164 primary prostate cancer samples from the TCGA dataset [22]. This RPPA analysis provides expression values of 188 epitopes (complete list in Supplementary Table S1), enabling us to test for correlations of these (phospho) proteins with mTOR-S2448 phosphorylation status (Figure 2A). Phosphorylation levels of PI3K pathway members, both upstream (p-AKT, p-TSC2) [10, 23] and downstream of mTOR (p-4EBP1, p-S6K, p-S6R) [10], positively correlate with p-mTOR. Targets that are suppressed by PI3K signaling, such as Bim, FOXO3a and IRS [24–27], show a negative correlation with p-mTOR expression (Supplementary Table S1). The correlation of p-mTOR with two downstream targets, p-4EBP1 and p-S6R, was validated with immunohistochemistry. Phospho-specificity of the antibodies was confirmed (Figure 2B–2C). Percentage of tumor cells with positive staining for phosphorylation of S6R (Ser240/244) and phosphorylation of 4EBP1 (Thr37/46) were scored on the same TMAs used for p-mTOR. Both p-S6R and p-4EBP1 positively correlated with p-mTOR (Spearman *r* = 0.355 (p < 0.0001), *r* = 0.265 (p < 0.0001) respectively). For additional analysis, patients were split by median percentage of positive tumor cells for each staining. Tissue with high p-mTOR staining also showed higher phosphorylation level of both S6R (p < 0.0001) and 4EBP1 (p = 0.012) (Table 4). Cumulatively, these data show that mTOR-S2448 phosphorylation is indicative for an activated mTOR pathway in prostate cancer, and this mTOR signaling pathway is activated specifically in prostate cancer patients with a favorable outcome.

**Table 4:** Comparison of p-mTOR expression with p-S6R and p-4EBP1

	Low p-mTOR	High p-mTOR	p-value
**p-S6R**			<0.0001
Low	72 (74%)	38 (42%)	
High	25 (26%)	52 (58%)	
**p-4EBP1**			0.012
Low	61 (64%)	40 (45%)	
High	34 (36%)	48 (55%)	

## DISCUSSION

Phase I/II clinical trials have shown limited efficacy of mTOR inhibitors in prostate cancer [16, 17]. This was a surprising outcome, since mTOR inhibitors proved successful in other malignancies, including breast and renal cancer [13, 14]. Two previous reports on the prognostic potential of mTOR S2448 phosphorylation in primary prostate adenocarcinoma showed conflicting results [18, 19], where p-mTOR did not associate with outcome in one [19], but correlated with good outcome in the other study [18].

Here, we report that high p-mTOR expression is associated with favorable outcome, which is in agreement with one of the two previous reports [18]. With this, we validated their findings despite the significantly smaller sample size of our study, stratifying patients on median (0-40% positive tumor cells) mTOR percentage as opposed to exclusively studying the minor p-mTOR-negative population (n = 9).

Furthermore we confirmed that the prognostic value of p-mTOR is limited to ERG positive cases. Still, a trend for good outcome was observed in the ERG-/p-mTOR+ population, implying that non-significant differences can be due to the relatively small sample size (n = 66). ERG expression alone does not have prognostic significance in this cohort [21] as reported by others [28].

It is unexpected that high p-mTOR, a marker of activated PI3K signaling, is associated with favorable prognosis in prostate cancer. Especially, since *in vitro* studies and PI3K pathway mutations in primary prostate cancer and mouse models implicate an oncogenic activation of PI3K signaling in prostate cancer [7, 29]. It is conceivable that mTOR phosphorylation in prostate cancer selectively plays a role in tumor onset and development rather than affecting disease progression. This potential role of mTOR activation in initial cell transformation as opposed to progression was also proposed in non small cell lung cancer [30] and intrahepatic cholangiocarcinomas [31], where mTOR activation was found in well-differentiated tumor cells.

Patients with high p-mTOR expression and mTOR pathway activation have a favorable prognosis and can be classified as low-risk for relapse, not requiring additional therapeutics beyond standard surgery and/or radiotherapy. Since high-risk patients have low mTOR activity, these patients may not benefit from mTOR inhibitors. Jointly, these results suggest no clear prostate cancer patient population exists that may benefit from mTOR inhibitor treatment. Future studies are aimed to assess whether these results can be confirmed in progressive disease and whether metastatic lesions have similar p-mTOR profiles.

In summary, phosphorylated mTOR, a marker of PI3K pathway activation, is associated with a favorable prognosis in primary prostate cancer. Prostate cancer patients with a high-risk of relapse have low-mTOR expressing tumors with an inactive mTOR pathway, and are consequently unlikely to benefit from mTOR inhibitor therapies. This provides a plausible explanation why mTOR inhibitors proved unsuccessful in prostate cancer trials.

## MATERIALS AND METHODS

### Immunohistochemistry

The prostate TMAs were previously described [21]. Tissues were stained for the expression of phosphorylated mTOR, S6R and 4EBP1 using a standardized protocol on the Ventana Benchmark® Ultra system automatic monostainer (Ventana Medical Systems). Details are provided in Supplementary Table S2. The percentage of tumor cells with positive staining was scored. Tissues scored for at least two cores were analysed, and the highest score was used for statistical analysis. The cut off for low and high p-mTOR expression is based on the median (Figure 1B). The ERG immunohistochemistry results on this cohort were previously reported [21].

For phosphatase treatment, tissue was incubated with 24000 units Lambda Phosphatase (sc-200312, Santa Cruz Biotechnologies) in 1× incubation buffer (supplied by Santa Cruz) for 2 hours at 37°C before applying the primary antibody. As control, a slide was incubated with only the incubation buffer without the Lambda phosphatase.

### Statistical analysis

Statistical relation between expression of p-mTOR (as categorical variable) and continuous clinico-pathological parameters (age and PSA at diagnosis) were tested using Student's *t*-test, and with categorical parameters (Gleason sum, pT-stage, surgical margins, ERG expression, p-S6R and p-4EBP1) using Pearson's x^2^ test. Highest scores of p-mTOR, p-S6R and p-4-EBP1 were used for calculation of Spearman correlation coefficients. Univariate and multivariate Cox regression were performed to evaluate the prognostic value of p-mTOR on biochemical recurrence. The covariates in the Cox regression model consist of two continuous variables (age and PSA) and five categorical variables (Gleason, pT stage, surgical margins, ERG and p-mTOR expression). A p-value of <0.05 was considered significant. All statistical analyses were performed using IBM SPSS Statistics version 22.

### RPPA

RPPA data from 164 primary prostate cancer samples generated by TCGA Research Network [22] was downloaded from the Cancer Proteome atlas website [32]. Pearson correlation analysis was performed between p-mTOR and protein levels for each protein represented in the RPPA data (list of proteins is provided in Supplementary Table S1). Volcano plot visualization is used to show the correlation coefficient versus the significance (adjusted for multiple testing).

### Western blotting

LNCaP cells were serum starved overnight and then pretreated with vehicle, 10 nM everolimus or 25 nM sirolimus for 3 hours followed by 100 ng/ml EGF for 20 min. The cells were lysed with Laemmli buffer supplemented with complete protease inhibitor cocktail and phosphatase inhibitors (NaF and sodium orthovanadate). Membranes were incubated with antibodies against p-mTOR S2448 (Cell Signaling Technologies, #2976) and actin (Millipore, MAB1501R).

## SUPPLEMENTARY TABLES




