# Supplementary material for: mTOR pathway activation is a favorable prognostic factor in human prostate adenocarcinoma
Source: Oncotarget. 2016 Apr 16;7(22):32916–24. doi: 10.18632/oncotarget.8767 (PMC5078062; doi:10.18632/oncotarget.8767)
Supplement: Supplementary file 1 [file oncotarget-07-32916-s001.pdf]

mTOR pathway activation is a favorable prognostic factor in human prostate adenocarcinoma

SUPPLEMENTARY TABLES

Supplementary Table S1: Correlation with mTOR-2448 phosphorylation (TCGA, RPPA data)

See Supplementary File 1

Supplementary Table S2: Antibodies and staining information for Ventana Benchmark® Ultra system automatic monostainer

Protein	phospho-site	Company	Cat. No.	Clone	Titer	antigen retrieval	primary
mTOR	Ser2448	Cell Signaling	2971	49F9	1:200	32 min CC-1 at 95°C	60 min at 36°C
S6R	Ser240/244	Cell Signaling	5364	D68F8	1:1000	32 min CC-1 at 95°C	32 min at 36°C
4EBP1	Thr37/46	Cell Signaling	2855	236B4	1:750	32 min CC-1 at 95°C	60 min at 36°C
